# Parenting Styles, Family Adaptation, and Cohesion in the Parental Families of Women with Borderline Personality Disorder (BPD) and Mentally Healthy Women

**DOI:** 10.1192/j.eurpsy.2026.11907

**Published:** 2026-07-31

**Authors:** V. I. Rozhdestvenskiy, V. S. Lupashku

**Affiliations:** Department of Clinical Psychology, Saint Petersburg State Pediatric Medical University, Saint Petersburg, Russian Federation

## Abstract

**Introduction:**

A number of studies confirm the link between dysfunctional parenting and the development of Borderline Personality Disorder **(**BPD) in women. Parents of women with BPD are often inconsistent in their upbringing and demonstrate a lack of emotional closeness with their daughters. However, the relationships between specific parenting styles and parameters of the family system—adaptation and cohesion—remain under-investigated.

**Objectives:**

The aim of this study was to identify the relationships between the levels of family adaptation and cohesion and the parenting styles of both parents in the parental families of women with BPD compared to the control group of mentally healthy women.

**Methods:**

Data were collected in 2025 using an online form developed by the authors. The sample consisted of 20 women diagnosed with Borderline Personality Disorder **(**BPD) and 20 women in the control group of mentally healthy women, aged 18–25. The Family Adaptation and Cohesion Scales (FACES-III) were used to assess family system parameters, and the Adolescents about Parents (ADOR) questionnaire was used to assess parenting styles. Both questionnaires were adapted for Russian-speaking respondents.

**Results:**

No statistically significant correlations were found between family adaptation, cohesion, and the parenting styles of both parents in the parental families of women with BPD. In the control group, family cohesion was positively correlated with maternal Closeness (r = 0.58, p < 0.01) and Positive Interest (r = 0.48, p < 0.05), and negatively correlated with maternal Hostility (r = -0.47, p < 0.05). Furthermore, family cohesion was positively correlated with paternal Closeness (r = 0.51, p < 0.05) and negatively with paternal Hostility (r = -0.45, p < 0.05). Family adaptation was negatively correlated with paternal Hostility (r = -0.64, p < 0.01), Directiveness (r = -0.45, p < 0.05), and Inconsistency (r = -0.53, p < 0.05), and positively correlated with paternal Closeness (r = 0.44, p < 0.05).

**Conclusions:**

The absence of significant links between family system parameters and parenting styles of both parents in the families of women with BPD may indicate profound dysfunction within the family system itself, or impairments in the perception and internalization of parental models by these women. In the control group, family adaptation and cohesion are clearly linked to specific parenting behaviors, reflecting more harmonious and integrated family relationships.

**Disclosure of Interest:**

None Declared

